# Age-Related Changes of Sprint Kinematics

**DOI:** 10.3389/fphys.2019.00613

**Published:** 2019-06-12

**Authors:** Julian Dahl, Hans Degens, Frank Hildebrand, Bergita Ganse

**Affiliations:** ^1^Department of Orthopaedic Trauma Surgery, RWTH Aachen University Hospital, Aachen, Germany; ^2^School of Healthcare Science, Research Center for Musculoskeletal Science and Sports Medicine, Manchester Metropolitan University, Manchester, United Kingdom; ^3^Institute of Sport Science and Innovations, Lithuanian Sports University, Kaunas, Lithuania; ^4^University of Medicine and Pharmacy of Târgu Mureș, Târgu Mureș, Romania

**Keywords:** aging, master athletics, track and field, running, video analysis, locomotion, age, sex

## Abstract

The sprint performance of master athletes decreases with age, but little is known about possible contributions of changes in sprint kinematics. The aim of this study was to assess the influence of age, sex and sprinting kinematics on sprint performance. To investigate this, in 199 men (30–89 years) and 81 women (33–76 years), bending over, brake, propulsion, leg stiffness and hip flexion angles were assessed during a sprint stride using high-resolution video analyses. Propulsion angle (men 25 ± 4.2, women 23.7 ± 4) was larger and hip flexion angle (men 25.3 ± 7.3, women 28 ± 5.7) was smaller in men than in women (both *p* < 0.001). Bending over angle (*p* = 0.004), brake angle (*p* = 0.004) and hip flexion angle (*p* < 0.001) increased, whereas propulsion angle (*p* < 0.001) and leg stiffness angle (*p* = 0.001) decreased with age, irrespective of sex. While performance was mainly determined by age (*R*^2^ = 0.501, *p* < 0.001) and sex (adjusted *R*^2^ = 0.642), hip flexion angle (adjusted *R*^2^ = 0.686) and bending over angle (adjusted *R*^2^ = 0.705) contributed also to performance in 60-m sprint. In 200-m sprint, in addition to age and sex, only hip flexion angle (age: *R*^2^ = 0.506; age + sex adjusted: *R*^2^ = 641; age + sex + hip flexion adjusted: *R*^2^ = 0.655) contributed to performance. In conclusion, the kinematics of sprinting differ between sexes and change with age. The aging-related changes of sprinting kinematics have a minor contribution to the aging-related decline in performance.

## Introduction

The aging society is a major challenge for Western countries in the twenty-first century. As the increasing life expectancy is not followed by an equal increase in healthy life years, the number of older people with morbidities increases (Jagger, 2015). This will not only put our healthcare systems to the test, but also emphasizes the need for interventions to increase health span (McPhee et al., 2016). Exercise is one of the major factors to maintain health (Kettunen et al., 2006) and mobility in old age. Indeed, physical activity reduces the risk of developing major cardiovascular and metabolic diseases, obesity, falls, cognitive impairments, osteoporosis, and muscle weakness (McPhee et al., 2016). Continuing sports with aging helps to maintain individual independence and reduces the need of acute and chronic care services (Shephard, 1993). While many older people are inactive because they fear incurring an injury, we have shown a low injury risk in healthy master athletes, which does not increase with age or performance (Ganse et al., 2014).

Track and field athletics encompasses different disciplines, including throwing and jumping, but the most popular are sprinting and middle- and long-distance running (Ganse et al., 2018a). The performance in running, sprinting (Ganse et al., 2018a) and javelin throwing (Ganse and Degens, 2018b) seems to show an accelerated decline after the age of 70 (Harridge and Lazarus, 2017). Success in sprint running requires a short reaction time, fast acceleration, high top velocity and high sprint endurance (Smirniotou et al., 2008).

Sprinting velocity is the product of stride rate and stride length (Dillman, 1975), and a high ground reaction force and a short ground contact time are associated with a good sprint performance (Weyand et al., 2000; Korhonen et al., 2003). Kinetic and kinematic parameters influence performance. Kinetic parameters include a loss of force generating capacity (Korhonen et al., 2005), slowing of the muscle, and increased tendon compliance during aging that all impair the ability to quickly develop high forces required for acceleration and maintenance of speed (Korhonen et al., 2006; Arampatzis et al., 2011). In line with this, an impaired ability to develop high forces quickly was associated with a longer ground contact time and reduced performance in older master sprinters (Korhonen et al., 2003, 2006). Changes in kinetics will have an impact on kinematic parameters (Van Caekenberghe et al., 2013), such as joint angles and body posture during the running cycle (Rabita et al., 2015), and these are the focus of the present study.

Lower forces in old age may result in a lower running speed due to a combination of the associated reduction of mechanical power output and a reduction in elastic energy storage during the landing phase for subsequent use in the propulsion phase, due to a reduction in leg stiffness (Pantoja et al., 2016). Indeed, high leg stiffness has been associated with a higher maximal sprinting velocity and faster acceleration (Bret et al., 2002).

For optimal energy transfer, the athlete needs to minimize the horizontal braking force, which may be realized by hitting the ground with the foot behind the body’s center of gravity (Harland and Steele, 1997). However, the age-related degeneration of bones and cartilage in the major joints (Boss and Seegmiller, 1981) may cause reductions in the range of motion due to pain and thus require altered running patterns that could further contribute to the reduced sprint performance in old age. For medical practitioners and scientists, knowledge on changes in motion patterns with age and how this may be related to fitness is important to define “healthy” motion patterns and plan exercise and rehabilitation programs to restore such patterns.

The objectives of this study were to assess (1) whether sprint kinematics changes throughout the aging process and (2) whether these changes contribute to the aging-related decline in sprint performance. As women have a lower muscle strength than men, similar aging-related changes and sex-related differences in running patterns may indicate that any such aging-related changes are an adaptation to lower muscle force/power. Therefore, (3) we also examined whether there were any sex-related differences in sprint kinematics.

The hypotheses of the study were that (1) sprint kinematics will change with age, (2) age-related changes in sprint kinematics will contribute to the finish time of master sprinters, and (3) there will be sex-related differences in sprint kinematics.

## Materials and Methods

Ethical approval was obtained from RWTH Aachen University Hospital IRB (reference number EK 300/17, date of approval: October 11, 2017). The IRB of RWTH Aachen University Hospital has decided that informed consent was not necessary for the present study, as data were only analyzed anonymously. Videos were recorded during a master athletics competition, and data were anonymized according to the Declaration of Helsinki.

### Subject Selection

All participants who successfully completed a 60-m and/or 200-m sprint at Nordrhein Westfalen master indoor track and field championships in Düsseldorf, Germany, on January 14th, 2018, were included in the study. Every athlete older than 30 who was registered with a track and field club in Nordrhein Westfalen and had an official start license could register for the event. There were no qualification standards, which means everyone could sign up, irrespective of their previous performance. The master athletes were allocated to the 5-year category groups common in master athletics, beginning at the age of 30 (30–34, 35–39, …, 80–84, 85–89).

### Materials

**Figure 1:**
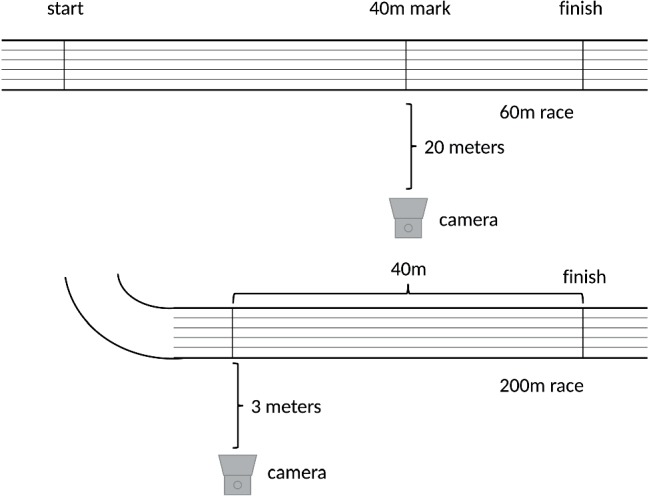
Camera locations.

Videos were recorded with a Nikon D3300 camera (Nikon, Düsseldorf, Germany), using a Nikon DX VR lens with a 55-mm focal length at 60 frames per second and a 1,080 pixel resolution. The camera, mounted on a tripod, was positioned as illustrated in Figure 1. The local setting of a standard indoor track and field stadium led to different distances between the camera and track for 60- and 200-m. This difference in position of the camera did not influence the analysis, as angles were only computed exactly orthogonally and with identical camera settings. The capture window was 4.8 m for the 200-m sprint and 12.5 m for the 60-m sprint.

### Software and Video Analysis

**Figure 2:**
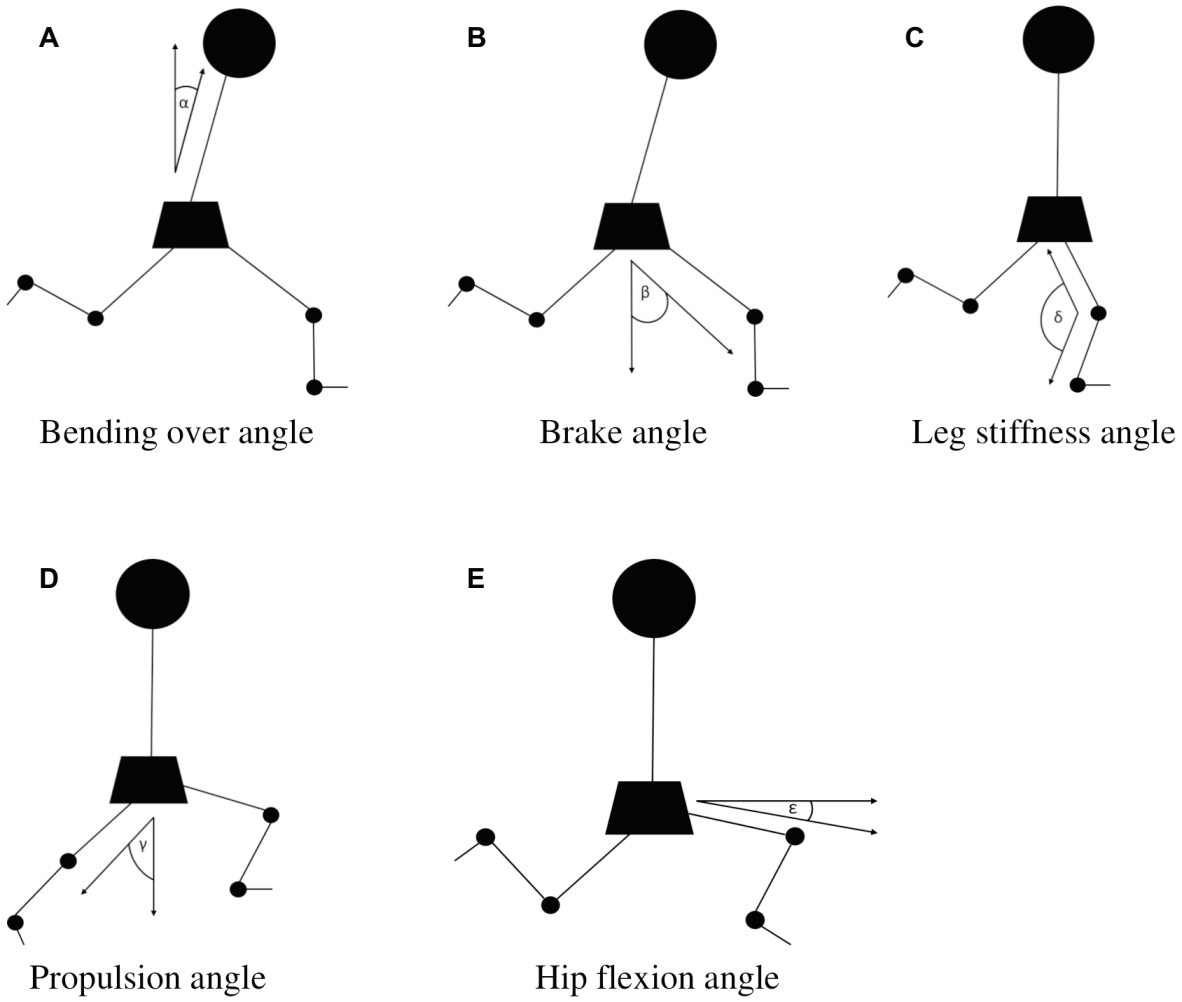
Description of angles. **(A)** *α:* bending over angle, phase 1 of the cycle of motion; **(B)** *β:* brake angle, phase 1 of the cycle of motion; **(C)** *δ:* leg stiffness angle, phase 3 of the cycle of motion; **(D)** *χ:* propulsion angle, phase 5 of the cycle of motion; and **(E)** *ε:* hip flexion angle, phase 6 of the cycle of motion.

Videos were analyzed using the open source video analysis software Kinovea version 0.8.15 (Joan Charmant & Contrib., France, Bordeaux), where every angle was measured by hand to reduce errors of measurement. Every stride during a sprint consists of two phases: the ground contact phase, where at least 1 ft of the sprinter is in contact with the ground, and the flight phase, where the sprinter has no contact with the ground. An individual stride can be described more detailed in seven phases as follows: (1) first contact of the foot with the ground in front of the hip, which is the beginning of the ground contact phase; (2) forward motion of the upper body until (3) the hip is directly above the foot; (4) force development of the grounded foot, while the upper body moves forward; (5) last contact of the foot with the ground; (6) beginning of the flight phase and flexion of the hip with the recovering leg, followed by (7) an extension until the next ground contact. For each athlete, five angles were computed as illustrated in Figure 2. The angles were measured in the following seven phases of the cycle of motion – phase 1: bending over angle (*α*), the angle between the upper body and the hips and brake angle (β), the angle between the line perpendicular to the ground and the line from the hip to the heel at first ground contact after the flight phase; phase 3: leg stiffness angle (δ), the knee angle when the hip was in a vertical line above the foot; phase 5: propulsion angle (*χ*), the angle between the line perpendicular to the ground and the stretched push-off leg at the end of the ground contact phase; phase 6: hip flexion angle (ε), minimal angle between a line through the hip parallel to the ground and a line from the hip to the patella in the flight phase. Angles were analyzed for the leg that was most orthogonal in view at the relevant moment of the running cycle.

Competition results and age data of the athletes were extracted from the track and field results databank Seltec^1^.

#### Statistical Analysis

All statistical tests were executed with IBM® SPSS® Statistics version 25. A two-way analysis of variance was applied to get information about sex differences and age effects on performance. Next, a three-way analysis of variance was conducted for every angle with the factors sex, race type and age to investigate if the angles differed between sex and race type and if they changed with age. If the angles showed a significant sex × age interaction, we performed a second ANOVA for men and women separately. Correlation and regression analyses were done for the angles and performance versus age to get information about aging-related changes in performance and the five angles. A stepwise linear regression was used to assess to what extent performance (performance as the race result [s]) was determined by age, sex, bending over angle, brake angle, propulsion angle, leg stiffness angle, and/or hip flexion angle. Only factors that correlated significantly with performance were fed into the model. Significance was assumed at *p* < 0.05. The data we used for statistical analysis can be found in the Figshare online database^2^.

## Results

In total, 280 races were filmed: 81 women (60-m races: 38; 200-m races: 43) and 199 men (60-m races: 112; 200-m races: 87). Athletes who performed in both disciplines were counted as one participant in each discipline (Table 1). Twelve participants (8 men in 60-m sprint, 3 women in 60-m sprint, and 1 woman in 200-m sprint) could not be included because they either did not reach the finish line or were disqualified. The oldest athlete in the study was 89 years. The average age of the participants was 54.2 ± 13.1 years.

**Table 1:** Number of participants in each discipline and age group.

Age group	Women 60-m	Women 200-m	Men 60-m	Men 200-m
30–34	4	2	5	1
35–39	3	4	13	8
40–44	7	6	4	7
45–49	8	9	14	12
50–54	5	9	19	18
55–59	5	5	19	11
60–64	2	3	7	4
65–69	2	3	12	9
70–74	2	1	6	6
75–79	0	1	6	6
80–84	0	0	4	4
85–89	0	0	3	1
Total	38	43	112	87

**Figure 3:**
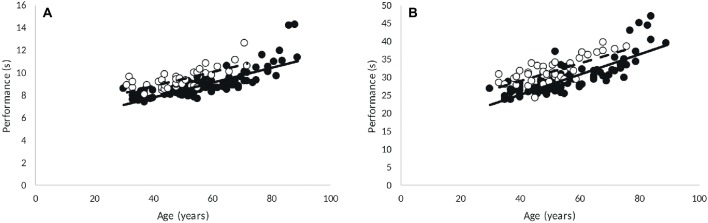
Correlations in 60-m **(A)** and 200-m **(B)** performance with age (men, 60-m: *R* = 0.801, *p* < 0.001; 200-m: *R* = 0.801, *p* < 0.001; women, 60-m: *R* = 0.727, *p* < 0.001; 200-m: *R* = 0.747, *p* < 0.001). Point: M, circle: F.

In Figure 3, it can be seen that men completed the 60- and 200-m sprint in a shorter time than women (*p* < 0.001). The absence of a significant age × sex interactions indicates that the aging-related decline in performance did not differ significantly between men and women.

### Impact of Discipline, Sex and Age on the Different Angles

**Figure 4:**
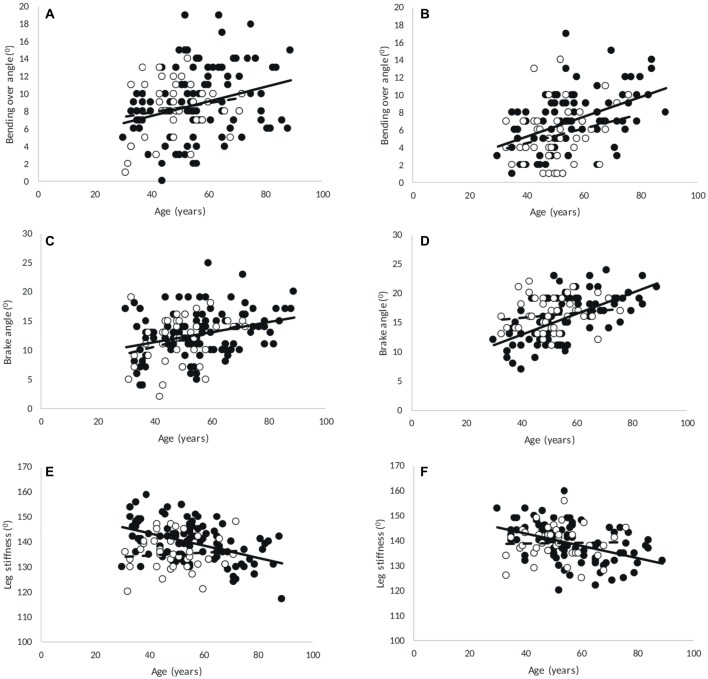
Correlations in 60-m **(A,C,E)** and 200-m **(B,D,F)** with age of **(A,B)** bending over angle (men and women, 60-m: *R* = 0.28, *p* = 0.001; 200-m: *R* = 0.424, *p* < 0.001), **(C,D)** brake angle (men and women, 60-m: *R* = 0.327, *p* < 0.001; 200-m: *R* = 0.494, *p* < 0.001), and **(E,F)** leg stiffness angle (men, 60-m: *R* = −0.447, *p* < 0.001; 200-m: *R* = −0.436, *p* < 0.001; women, 60-m and 200-m: not significant). Point: M, circle: F.

Bending over angle was smaller and brake angle was larger in the 200- than 60-m sprint (*p* < 0.001), and both increased with age (*p* = 0.004), irrespective of sex and discipline (Figure 4).

The leg stiffness angle did not differ significantly between 60- and 200-m sprints. Although there was a significant age × sex interaction (*p* = 0.003), in both men (*p* < 0.001) and women (*p* = 0.025) the leg stiffness angle decreased with age, irrespective of discipline (Figure 4).

**Figure 5:**
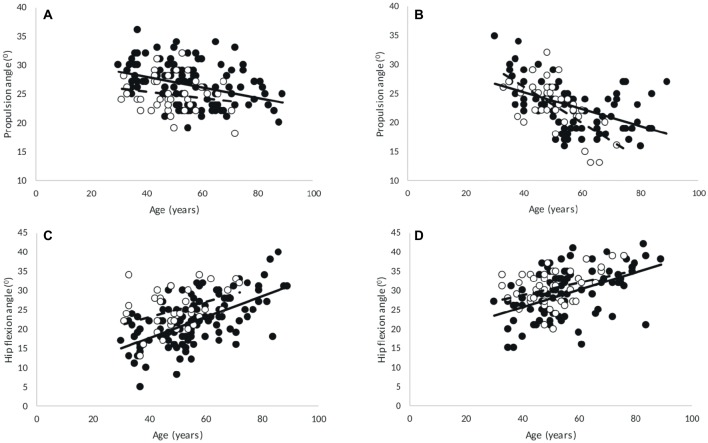
Correlations in 60-m **(A,C)** and 200-m **(B,D)** with age of **(A,B)** propulsion angle (men, 60-m: *R* = −0.35, *p* < 0.001; 200-m: *R* = −0.485, *p* < 0.001; women, 60-m: no significances; 200-m: *R* = −0.694, *p* < 0.001), and **(C,D)** hip flexion (men, 60-m: *R* = 0.604, *p* < 0.001; 200-m: *R* = 0.489, *p* < 0.001; women, 60-m: *R* = 0.394, *p* = 0.014; 200-m: *R* = 0.36, *p* = 0.018). Point: M, circle: F.

There were main effects of discipline, age and sex for propulsion angle (all *p* < 0.001). The propulsion angle was smaller in 200- than in 60-m races. There was a significant sex × age interaction for propulsion angle (*p* = 0.024), but *post hoc* analysis showed that the propulsion angle declined with age in both men (*p* < 0.001) and women (*p* = 0.001), irrespective of discipline (Figure 5).

Hip flexion angle was smaller in the 60-m sprint than in the 200-m sprint (*p* < 0.001) and higher in women than in men (*p* < 0.001). The hip flexion angle increased with age (*p* < 0.001), irrespective of discipline and sex (Figure 5).

### Determinants of Sprint Performance

A stepwise regression for the 60-m sprint showed that age was the most important predictor of performance (*R*^2^ = 0.501, *p* < 0.001), with a contribution of sex (adjusted *R*^2^ = 0.642), hip flexion (adjusted *R*^2^ = 0.686) and bending over angle (adjusted *R*^2^ = 0.705). For the 200-m sprint, age was also the most important factor (*R*^2^ = 0.506, *p* < 0.001), followed by sex (adjusted *R*^2^ = 0.641) and hip flexion (adjusted *R*^2^ = 0.655). Brake angle, propulsion angle, and leg stiffness angle did not influence the performance significantly.

## Discussion

The main observation of the present study is that all angles change throughout the aging process. However, the similar aging-related decline in sprint performance in men and women is only to a small degree associated with changes in running kinematics. We propose here that the change in running kinematics is not so much a cause of the decline in running performance, but rather an adaptation to the muscle weakening in old age. For instance, (1) a lower hip flexion angle is also seen in women (who have less muscle power) than men and (2) higher brake angles are also seen at the end of the 200-m than at the end of the 60-m race, presumable due to larger muscle fatigue during the 200-m run.

### Effects of Age

In line with our previous work, sprinting performance decreased with age (Ganse et al., 2018a). It has been shown that lower performance in older than younger athletes was attributable to a lower stride length and flight time, and increased ground contact time in older athletes (Korhonen et al., 2003). Part of these changes in stride length may be due to a decrease in the range of motion of hip and knee joints with age (Hamilton, 1993) that may contribute to the larger hip flexion and smaller leg stiffness angles we observed in the older athletes. As sprinting kinematics rely on a cycle of motion, the change of one parameter will likely cause a series of reactions where, for instance, it has been observed that delayed peak joint angle timing and angular velocity parameters occurred during the gait cycle (Afiah et al., 2016). The increase in ground contact time with age (Korhonen et al., 2003) was associated with weaker muscles that also were less able to generate force rapidly (Kohornen et al., 2009). The significance of muscle weakness as a cause of the aging-related changes in sprint kinematics is further supported by our observation that the aging-related increase in brake angles is mimicked by the larger angles in the 200- than 60-m sprint, probably due to larger muscle fatigue in the 200-m sprint. These observations thus suggest that the main cause of the aging-related difference in running pattern is most likely the loss of force and power generating capacity of the muscles, with probable contributions of chronic diseases and pain.

### Determinants of Sprint Performance

Age was the major predictor of performance, followed by sex, while kinematics only had a minor effect in the present study. Although Ito et al. (2008) found in elite sprinters that a high hip flexion is not as important as previously assumed, the general observation is that hip flexion correlates positively with running performance (Copaver et al., 2012; Haugen et al., 2018). In line with this, Dillman et al. observed that highly skilled runners tend to lift the thigh higher (so smaller hip flexion angle) during the flight phase compared to less skilled athletes (Dillman, 1975). We found a contribution of hip flexion to performance (60-m: *R* = 0.64, *p* < 0.001; 200-m: *R* = 0.51, *p* < 0.001; note that this positive correlation means that the smaller the angle the shorter the run and hence the better performance). What these correlations do potentially obscure, however, is the role of muscle weakness during aging in adapting the motion pattern, and indeed as discussed above, the hip flexion angle increased with increasing age. One way to overcome this potential bias is to perform a stepwise regression, which revealed that age is the main predictor of performance (around 51% of the variation in performance), with an additional contribution of sex, and only 6% explained by differences in the hip flexion and bending over angles.

### Sex Differences

We found that on average men completed the 60- and 200-m in a shorter time than women. This is in line with the results of other studies, where men were faster than women, especially in shorter distances (Nikolaidis et al., 2017; Schneider et al., 2018). As power output is an important determinant of performance in a 100-m sprint (Slawinski et al., 2017), the larger power in men than women (Perez-Gomez et al., 2008) may well be the explanation of this difference in performance between men and women. In addition, differences in sprint kinematics between men and women may contribute, where women showed a higher peak plantar flexion and range of motion in the sagittal plane (Takabayashi et al., 2017) and a higher knee abduction and hip adduction than men (Phinyomark et al., 2016), which may contribute to the larger hip flexion angle we observed in women. Women are affected more frequently by foot injuries (Phinyomark et al., 2016) and anterior cruciate ligament injuries (James et al., 2004), which is suggested to be a result of differences in motion patterns. We suggest here that these sex differences in kinematics are an adaptation to the differences in muscle strength between men and women, as aging that is associated with muscle weakening is also associated with an increase in hip flexion angle during the sprints.

### Limitations

Although sprinting is performed in three-dimensional space, the angles we measured are all in one plane, where less happens biomechanically in the frontal plane. We therefore contend that missing the frontal plane will not significantly affect the outcome of our study. While we measured relatively few women older than 65 years, a clear aging-related decrement in performance was observed in our population. It may be that aging-related changes in angles would have been more pronounced in older individuals and have shown an accelerated change beyond the age of 70 years, but the data we have of people older than 70 years for male sprinters suggest that no such acceleration occurs. Another limitation is the cross-sectional study design. In addition, we only included kinematic and no kinetic measurements.

### Practical Applications for Practitioners

Hip flexion and bending of the upper body were the only two kinematic parameters that, apart from aging, showed some influence on performance. Master sprinters may therefore benefit from increasing their hip range of motion by increasing the power of the hip flexors and trunk muscles. The same applies to geriatric physiotherapy and fitness programs, where such interventions may lead to improvement of walking ability and hence quality of life.

## Conclusion

Here, we found that age was the major predictor of performance, followed by sex. The decline in sprint performance during aging was only to a small extent attributable to aging-related changes in kinematics, such as increased hip flexion and bending over angles. The observed aging-related changes in running kinematics are most likely adaptations to the lower muscle force and power generating capacity in older athletes. The lower hip flexion in women than men contributed somewhat to the lower sprint performance in women than men. Future studies may explore to what extent differences in running kinematics in muscle strength- and power-matched individuals contributes to sprint performance and whether running kinematics may change after a period of strength training exercise and improve performance.

## Ethics Statement

Ethical approval was obtained from RWTH Aachen University Hospital IRB (reference number EK 300/17, date of approval: October 11, 2017). Informed consent was not needed.

## Author Contributions

JD contributed to data collection, data analysis, interpretation, figures, tables, manuscript drafting, and approval of the manuscript. HD worked on data interpretation, statistical analysis, drafting, and approval of the manuscript. FH supported with data interpretation and approval of the manuscript. BG contributed to the idea and worked on data interpretation, drafting, manuscript submission, and approval of the manuscript.

### Conflict of Interest Statement

The authors declare that the research was conducted in the absence of any commercial or financial relationships that could be construed as a potential conflict of interest.

## Footnotes

^1^https://laportal.net/Competitions/Details/198

^2^https://figshare.com/s/9b7d2280a0671b4076fe
